# Plain Speaking on Tuberculosis Matters

**Published:** 1920-07-31

**Authors:** 


### PLAIN SPEAKING ON TUBERCULOSIS MATTERS.

For years the local newspapers of the country, north, south, east and west, have been printing accounts of troubled committee meetings dealing with complaining patients, runaway patients, unsatisfactory results, increasing disease, depletion of funds, want of harmony among the staff, and so on. But, on the other hand, these regrettable phenomena seem to find no recognition other than local. The spokesmen for the British Anti-tuberculosis Campaign (most of them, at least) seem to have no knowledge of any such grave faults. Now the policy of putting a good face on things at whatever cost is a policy that can be overdone, as the aftermath of that other and greater war now abundantly shows. This is a time, for very many people, of the beginning of an utter disillusionment, of a process of extricating themselves from Whistler's category of those "misled by an overwhelming conviction of right." Even the yellow press is reluctantly coming to see that it will no longer do to assert, directly counter to facts, that the Germans copy us or the French, or that this country has a monopoly of virtue. Equally inexpedient is it to go on hushing up the unsatisfactory state and tendency of the British Anti-tuberculosis Campaign.

This last view seems to be fully shared by a writer whose production,* a pamphlet of thirty-eight succinctly written pages, is now before us. He begins by emphasising the important point, manifest from war experience, especially Continental war experience—and first mentioned in this country in a leading article in the *Lancet* (to the fluttering of certain Northern dovecots)—that anti-infection measures have been shown to be less, and the maintenance of individual constitutional resistance far more, effective than was thought in 1914. Passing rapidly from this, however, he explains that it is not so much the plan of campaign that is impugned, as faults of execution that would make nugatory any plan. And writing from experience, he arranges his criticism under two main heads—the management of public sanatoriums, and the election of tuberculosis officers. As regards the first, it is shown that the running of British public sanatoriums on the hospital system, with visiting honoraries, matron and elaborate nursing personnel, secretary, perhaps chaplain, and so on, is completely counter to the precepts and practice of such acknowledged pioneers and authorities as Brehmer, Walther, Bodington, Trudean, and Paterson. It leads to the nullification of the most important resident official, the medical superintendent, and puts the treatment of convalescent consumptives, "mostly young, full of *spes phthisica*, and fed like fighting cocks," on a level with that of the acutely ill and mentally depressed hospital patient. Some striking examples are given of the almost kaleidoscopic change in the tenure of the above posts, and of the ruinous mismanagement of a public sanatorium, leading to intervention by the Local Government Board. Centralised control of anti-tuberculosis measures is desiderated, as recommended by the late Sir William Osler, with co-operation by neighbouring sanitary authorities in the maintenance of one large and therefore economical sanatorium, where facilities could be provided for the employment of ex-patients, and where, in assistant posts, medical men could be trained for the tuberculosis service.

In the matter of tuberculosis officers, the author's experience has caused him grave doubts as to the fairness of election to public-health appointments, lack of experience, with no countervailing merit, having been repeatedly allowed to triumph over its presence. The final chapter of constructive criticism may be deduced from what has gone before.

Much of this indictment sounds with the ring of truth and of *choses vues*. It comes, the author remarks, at a good time, when the laity is beginning to question the value of the current anti-tuberculosis movement, as judged by its results. If abuses are rectified, then an improvement is to be expected. The next step is full recognition of the point we began with, that freedom from tuberculosis is more a matter of general physiological fitness than of avoidance of infection: war tuberculosis came far too suddenly, far too widely, far too independently of infection, far too closely connected with hardship and privation, for anything else to be possible. And the last—last because entailing political change, and not medical and philanthropic effort merely, but first in practical prophylactic importance—is attention to the sociological aspect of the disease. Now that the teachings of hygiene are well known, those able to follow them are showing an increasing freedom from tuberculosis. What is indicated most plainly is social amelioration, the raising of the working classes to a similar physically and mentally and morally healthy level. The beginnings of this process are now rapidly passing out of the initial stage. In no insignificant part are they, in all probability, destined to be (as Pater said of the universalisation of a certain shade of character) "the redemption of the world."

* *Anti-Tuberculosis Reconstruction.* By D.P.H.R.C.P.S.I. Maunsel & Co., Lower Baggot Street, Dublin. 6d. net.

